# Extra-Uterine Pregnancy

**Published:** 1892-06

**Authors:** A. E. Aust Lawrence

**Affiliations:** Physician Accoucheur to the Bristol General Hospital, Lecturer on Midwifery and Diseases of Women at the Bristol Medical School


THE BRISTOL

### Medico-Chirurgical Journal.

JUNE, 1892.

### EXTRA-UTERINE PREGNANCY.

BY

A. E. Aust Lawrence, M.D., C.M. Aberd.,

*Physician Accoucheur to the Bristol General Hospital, Lecturer on Midwifery and Diseases of Women at the Bristol Medical School.*

My desire in this paper is to present, as briefly and in as practical a manner as I can, the very important subject of Extra-uterine Pregnancy, or, as it is sometimes called, Ectopic Gestation.

In a communication like this there is no need to discuss the anatomy, physiology, or pathology of the subject. These points are all set forth in many well-known and excellent handbooks. It matters very little to a woman whether the ovum is impregnated in her ovary, her Fallopian tube, or her uterus, but it does matter to her where the ovum develops. For all practical purposes the abnormal site of development to be considered is the Fallopian tube, and, with very few exceptions, this is the seat of the pregnancy, which we may have to treat under the heading of extra-uterine gestation.

To facilitate consideration of the subject, I have made four classes of cases:

1. Cases in which nothing can be done. The first intimation of anything abnormal existing in these cases is the sudden onset of alarming symptoms, such as abdominal pain, collapse, and death. The *post mortem* examination reveals a Fallopian tube distended and burst, with free hemorrhage into the abdomen. I have not yet met with a case of this description, but there have been many recorded. The patient dies in a very few minutes, or, at the most, in an hour or two.

2. Cases in which the patient survives the primary rupture, and in which the ovum escapes from the Fallopian tube and develops in the abdominal cavity. Two of my cases are examples of this kind.

3. Cases in which the patient does not die immediately, but gets repeated attacks of pain, &c., due to repeated bleedings from the ruptured tube or fœtal structures. Three of my cases are examples of this variety.

4. Cases in which all the evidence points to ectopic gestation, but in which serious symptoms do not occur more than once, from the fact that with the primary rupture of the tube and escape of blood either into the broad ligament or free into the abdomen the ovum perishes, and the hemorrhage does not return; or there may be only distension of the tube with blood and no rupture. These cases require no active treatment. I am sure I have seen several cases of arrested ectopic gestation recover completely without operation. It is most important to be able to distinguish between this set of cases and the set in which repeated bleedings do occur, and the patient's chance of recovery hangs on the diagnosis.

I shall now briefly relate my cases, and then emphasise the important points which help us to diagnose—first, the nature of the case, and, secondly, the class of case.

E. R., aged 27, came to my out-patient room in the Bristol General Hospital, in January, 1888, complaining of great abdominal and pelvic pains, from which she had suffered for twelve months. She had missed one period at this date, and was suddenly taken ill with severe pain in the abdomen and faintness. I found an irregular tumour lying across the lower abdomen, and it could be felt *per vaginam;* and through the rectal wall I distinctly felt a foot of a fœtus free in Douglas' pouch.

I opened the abdomen, and removed a six months' fœtus which had been dead some months. The fœtus was free in the abdominal cavity. I brought the umbilical cord out of the wound, leaving the placenta untouched at the back of the uterus. The cord came away at the end of ten days; the wound closed at the end of a month; the placenta became absorbed, for at the end of six months I examined her, and found no trace of the placenta, which had been distinctly felt as a large retro-uterine swelling.

C. D., aged 31. I saw this patient in the country. She went to her full time with a living child in her abdomen, and apparently ordinary labour set in; the os uteri dilated in the usual way, and it was only at this stage that her real condition was discovered. During the whole of her gestation she had general abdominal pains, but there was no definite history of the usual severe symptoms indicating primary rupture of the tube. This may not have occurred. I saw her late one evening, and just before

7 *

my arrival the child ceased to move and died. As the child was dead, I advised, under the circumstances, that she should be kept in bed for two or three weeks and then brought into the hospital. Had the child been alive, I should have operated at once; but as it was dead, I deemed it wise to give time for the placental circulation to lessen. The patient was brought into the hospital, and I opened her abdomen, and found a full-term child free in the peritoneal cavity. I removed it, and fixed the end of the cord outside the abdomen. At the end of fourteen days the cord came away, and the wound healed. At the end of the month, as the temperature began to rise, the wound was re-opened, and a piece of placenta came away. I passed my finger down the sinus, which I had well washed out with boracic lotion. The discharge, in spite of all, became very offensive, and the patient died of septicæmia. The *post mortem* examination showed the remaining part of the placenta to be loose in a sac formed by inflammatory adhesions of intestines, &c.

From the consideration of these two cases, together with one of Mr. Greig Smith's and one of Dr. Champneys', I am inclined to say that the proper treatment of the placenta in these abdominal gestation cases (that is, where it cannot be removed as part of a gestation sac) is to tie the cord close down to the placenta, clean out the abdominal cavity, insert a glass drainage-tube for a day or two, and then let the wound close. The placenta may give no further trouble, but be absorbed, as it was in my case, and I believe in Mr. Greig Smith's case on which he operated about the same time that I did. But if symptoms of septic trouble arise, the abdomen should be opened again, and the placenta removed if possible. In my case, it might have saved the woman, and I think also in Dr. Champneys' case, which ran a course very similar to my own. We all know the great danger of attempting to separate the placenta consists in the hemorrhage; and cases have been recorded where, after some months, attempts to remove the placenta have been followed by death from hemorrhage. But I consider we should explore if septic symptoms set in, and, if possible, remove the placenta, or, at all events, well drain that portion of the abdominal cavity where it is situated.

The three cases remaining were all examples of the class of early rupture of the tube and hemorrhage occurring at intervals.

The first (R. L.) was brought into the hospital with the history of having missed one period, and having been taken with great pain in the abdomen and faintness two days before. On her way in she had attacks of violent pain. I suspected extra-uterine gestation, and opened her abdomen. I found the diagnosis was correct; but all the parts were so matted together that I could not remove the damaged organs, and she died a few days after the operation. The *post mortem* examination revealed the exact nature of the case, and the specimen I showed at the Bristol Medico-Chirurgical Society last year.

The fourth case (S. W., aged 24) illustrates the great advantage of early consultation. This woman missed one period, and when the second should have come on she was taken with severe abdominal pain and faintness, but in a few hours she appeared quite well. She was, however, sent into the hospital. I examined her, and found a swelling as large as a small hen's egg to the left of the uterus. I suspected Fallopian gestation, and arranged, if any severe symptoms occurred, that I would open the abdomen.

During the night severe pains in the abdomen and faintness set in. The next morning she appeared pretty well. I found the swelling to the left of the uterus had disappeared, and instead of it there was a general fulness behind and at both sides of the uterus, and the lower abdomen also had a distinct line of resistance for a couple of inches above the pubes, and extending laterally to the iliac spines. I felt sure I was justified in opening the abdomen, and did so, and found at least a quart of blood free and the ruptured tube. I removed the tube, washed out the abdomen, and drained it for three weeks, as broken-down blood-clot came away daily. She made a perfect recovery. I consider that drainage is most important in these cases. Her first symptoms were due to sudden distension of, and *not* rupture of, the tube; the next attack of pain, &c., was coincident with the rupture of the tube and escape of blood into her abdomen.

Before I saw the case that I have just related, I saw, with a medical friend, a similar one, and it was the experience I gained from that case that helped me to diagnose my own; and it was the knowledge I gained by watching Mr. Greig Smith operate on that patient that enabled me to operate on my own case with such good results. That case began, like mine, with a swelling at the side of the uterus; but when I saw the patient this had disappeared, and I found a localised swelling behind the uterus (not at the sides of the pelvis), the uterus pushed up against the pubes, the os soft. I made an error in the diagnosis, and considered that it was a case of retroflexion of an early gravid uterus. Two days after I saw her again, and then the retro-uterine swelling had extended to the sides of the pelvis; and I then suspected extra-uterine gestation, with ruptured Fallopian tube and free bleeding into the abdomen. Mr. Greig Smith then saw the case with us, and agreed in the suspicion; but as the patient's condition was fairly good, and the diagnosis doubtful, we agreed to wait. A further hemorrhage took place, and the patient was operated on. I simply mention the case as it shows how very easy it is to mistake a retro-uterine hemorrhage, with displacement forward of the uterus, for a retroflexed gravid uterus. I have seen and treated at least 50 cases of retroflexion of the gravid uterus, and yet, in spite of that, I mistook the nature of the case. I did not attach enough importance to the fact mentioned to me, that there was, to begin with, a left lateral swelling.

Previous to my last case, I saw, in consultation with another medical friend, a case with the history of suspected pregnancy and recurring attacks of pain in the abdomen, faintness, &c. When I saw the patient she was very pale, and the abdomen swollen. I diagnosed ruptured tubal pregnancy, and advised operation, which was done and the diagnosis confirmed, the abdomen being full of blood.

The fifth case I believe to be one of ectopic gestation. The woman had missed one period, and thought herself pregnant. She was suddenly seized with abdominal pain and faintness. There was distinct evidence of a large hæmatocele, both *per vaginam* and by abdominal examination. I kept her quiet a few days, and then she had a very severe attack of pain and her temperature ran up. I opened her abdomen, and found recent peritonitis with effusion, and a small black blood-clot in the fluid. I suspected at once leakage of an encysted hæmatocele, probably decomposing. With great difficulty, I broke through the roof of the hæmatocele

(formed by intestines matted together and fixed to the uterus, bladder, &c.), and evacuated about two pints of blood and blood-clot. I removed the tube and the ovary, from the surface of which free bleeding was going on, owing to the adherent intestines, &c., being torn from it. I broke away, in fact, a piece of ovarian capsule, with, I believe, the fimbriated end of the Fallopian tube attached to it, forming a gestation sac. I drained the abdomen for ten days, and she made a perfect recovery.[1] Whether this was originally an ectopic gestation which caused the hemorrhage or not, I cannot be absolutely sure; but I consider it was, and most likely a tubal-ovarian gestation. Under any circumstances, I consider that when we have a large hæmatocele showing signs of becoming purulent by rise of temperature, &c., it is our duty to open, wash out, and drain the cavity.

Shortly after this case I had another, where there was a suspicious history of pregnancy and then sudden abdominal pain and faintness, and for days persistent pain and high temperature. I suspected ectopic gestation, and opened the abdomen and found a large firm swelling, reaching from the brim of the pelvis half-way up to the umbilicus, consisting of bladder and intestines and effused products of inflammation. No uterus, ovaries, or tubes could be found; and in spite of persistent endeavours to open the swelling, I could not succeed. Two days after the operation, the patient passed *per rectum* a large quantity of broken-down blood or pus, and died after ten days with symptoms of septicæmia. The *post mortem* examination showed a very large cavity behind the uterus and one tube, which Mr. Morton examined and

[1] Mr. Morton kindly made an examination of the specimens in these two last cases.

considered to give evidence of having been the seat of ectopic gestation.

The points which help us in the diagnosis of tubal pregnancy may be thus summarised:

1. *History of the Patient.*—Often there has been for some years sterility, pointing most likely to tubal disease. There is the history of supposed pregnancy, on account of a period having been missed. But if no period has been missed, it must not be concluded that pregnancy does not exist; for in cases of tubal gestation, particularly where the tube is diseased, and even in normal uterine pregnancy there may be a periodic uterine hemorrhage, and, therefore, the non-missing of a period must not be held as conclusive of the absence of gestation. It is most important to get as accurate a history as possible from the patient or her friends.

A sudden attack of abdominal pain, faintness, and sickness, occurring in a woman who has had no definite local ailment which might have given rise to the symptoms, must be considered serious. If this sudden attack has been preceded by the missing of one or more periods, it is gravely suspicious of ectopic gestation; and if there is a recurrence of the attack in a few days or a week or two, it is still more suspicious.

2. *The Examination of the Patient.*—The woman may be, or may not be, very anæmic: this depends on the loss of blood, or on the recent or late occurrence of her primary attack. A woman with a large effusion of blood into the abdomen would be pale at whatever stage she was seen; but a woman may be very pale and collapsed with only a very slight loss of blood, simply from shock, as occurred in one of my cases where simple distension of the tube without rupture caused collapse.

The abdominal examination helps to a certain extent, especially where the effusion of blood is great. A swelling is recognised as coming up from the pelvis to a varying height in the abdomen; and if there is any sudden increase of this swelling, with the symptoms of pain, &c., it is very suspicious of fresh bleeding. Of course, all bladder complications must be excluded, and, by the history, all abdominal disease previous to the onset of the present symptoms.

A good deal can be learned from a vaginal examination, but it is almost impossible even for the most experienced to diagnose the case simply on this examination alone. I have already mentioned the case where I concluded I had to deal with a retroflexed uterus, and many cases at the outset have been regarded in this way. The great thing to do is, not to try to make too exact a diagnosis. If the mass behind the cervix uteri seems to be the fundus (and in some cases it feels rounded and continuous with the cervix uteri), a change of position may remedy the displacement if it exists. If there is a very painful swelling by the side of the uterus, and if in a few days this is lost in a general pelvic swelling with acute symptoms, it may be assumed that there is a ruptured tube, with or without pregnancy.

The most difficult case I have had to diagnose lately was that of a lady who came to Clifton to be under my care. She had a history of missing two periods. She had a rounded swelling to the left side of the uterus, and one posterior to the uterus; the os was soft. She also had had two or three attacks of uterine hemorrhage. I thought it was retroflexion of the gravid uterus, with diseased ovary and tube fixed to the side of the uterus; but I could not say that it was not a retroflexed nonpregnant uterus with a Fallopian gestation. I was helped in the diagnosis by the absence of anything like severe pain or shock, which she must have had if the swelling by the side of the uterus was a pregnant tube; for she must have had pain from the distension of the tube, although it may not have been ruptured. Another point in her history helped me. She had been under my care some years before, with chronic pelvic pains. I made her assume the left lateral position, and in the course of a week the fundus uteri resumed its normal position, and the lateral swelling remained for two months as it was when I first saw her, and I have no doubt it was a chronic inflammatory condition of the left tube and ovary. Her pregnancy was uterine, and progressed normally.

One fact relied on is the passing of a cast of the uterus. This is generally coincident with the cessation of the abnormal pregnancy. The uterus when pregnancy takes place forms the usual decidual lining, and when the pregnancy is interrupted it casts off this lining; but by itself it is not a trustworthy sign, as it is so liable to be mistaken for early abortion, and in consequence the gravity of the case is entirely overlooked.

The whole diagnosis may be summed up in a few words. I should say that when a previously healthy woman misses one or more periods and is taken with acute abdominal pain and fainting, and these symptoms recur at short intervals, and the vaginal examination reveals a retro- and peri-uterine hæmatocele, either extending or not up into the abdomen, it is imperative to open that abdomen without delay.

